# The molecular machinery of regulated cell death

**DOI:** 10.1038/s41422-019-0164-5

**Published:** 2019-04-04

**Authors:** Daolin Tang, Rui Kang, Tom Vanden Berghe, Peter Vandenabeele, Guido Kroemer

**Affiliations:** 1 The Third Affiliated Hospital, Protein Modification and Degradation Lab, School of Basic Medical Sciences, Guangzhou Medical University, 510510 Guangzhou, Guangdong China; 2 Department of Surgery, UT Southwestern Medical Center, Dallas, TX 75390 USA; 3 Molecular Signaling and Cell Death Unit, VIB-UGent Center for Inflammation Research, Flanders Institute for Biotechnology, 9052 Ghent, Belgium; 4 Department for Biomedical Molecular Biology, Ghent University, 9052 Ghent, Belgium; 5 Laboratory of Pathophysiology, Faculty of Biomedical Sciences, University of Antwerp, 2610 Wilrijk, Belgium; 6 Methusalem program, Ghent University, 9000 Ghent, Belgium; 7 Université Paris Descartes, Sorbonne Paris Cité, 75006 Paris, France; 8 Equipe 11 labellisée Ligue Nationale contre le Cancer, Centre de Recherche des Cordeliers, 75006 Paris, France; 9 Institut National de la Santé et de la Recherche Médicale, U1138 Paris, France; 10 Université Pierre et Marie Curie, 75006 Paris, France; 11 Metabolomics and Cell Biology Platforms, Gustave Roussy Cancer Campus, 94800 Villejuif, France; 12 Pôle de Biologie, Hôpital Européen Georges Pompidou, AP-HP, 75015 Paris, France; 13 Department of Women’s and Children’s Health, Karolinska University Hospital, 17176 Stockholm, Sweden

**Keywords:** Cell signalling, Cell death

## Abstract

Cells may die from accidental cell death (ACD) or regulated cell death (RCD). ACD is a biologically uncontrolled process, whereas RCD involves tightly structured signaling cascades and molecularly defined effector mechanisms. A growing number of novel non-apoptotic forms of RCD have been identified and are increasingly being implicated in various human pathologies. Here, we critically review the current state of the art regarding non-apoptotic types of RCD, including necroptosis, pyroptosis, ferroptosis, entotic cell death, netotic cell death, parthanatos, lysosome-dependent cell death, autophagy-dependent cell death, alkaliptosis and oxeiptosis. The in-depth comprehension of each of these lethal subroutines and their intercellular consequences may uncover novel therapeutic targets for the avoidance of pathogenic cell loss.

## Introduction

**Fig. 1:**
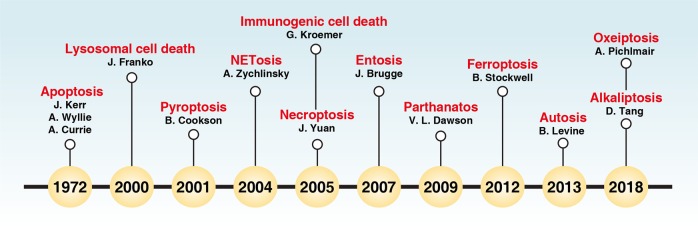
Timeline of the terms used in cell death research

**Fig. 2:**
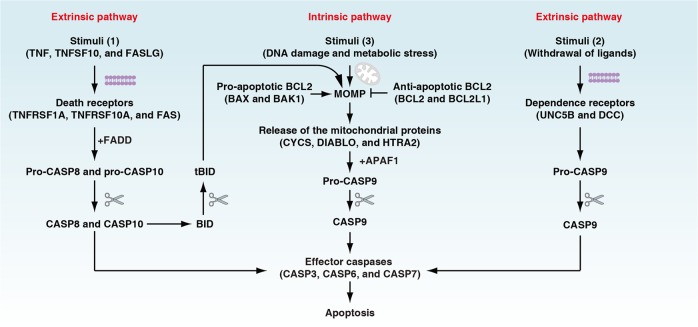
Extrinsic and intrinsic apoptosis. Extrinsic apoptosis is induced by the addition of death receptor ligands or by the withdrawal of dependence receptor ligands. CASP8 and CASP10 initiate death receptor-mediated extrinsic apoptosis, whereas CASP9 initiates the withdrawal of dependence receptor ligand-mediated extrinsic apoptosis. Pro-CASP8 and pro-CASP10 are enzymatically inactive until they interact with FADD (Fas-associated via death domain), which is activated upon binding to cell death receptors responding to their ligands. DNA damage, hypoxia, metabolic stress, and other factors can induce intrinsic apoptosis, which begins with MOMP and leads to the release of mitochondrial proteins (e.g., CYCS) into the cytosol. Cytosolic CYCS interacts with APAF1, which recruits pro-CASP9 to form the apoptosome. MOMP is tightly controlled by the BCL2 family, including its pro-apoptotic and anti-apoptotic members. CASP3, CASP6, and CASP7 are considered the common effector caspases for both extrinsic and intrinsic apoptosis. In addition, the extrinsic pathway can trigger intrinsic mitochondrial apoptosis through the generation of truncated BID (tBID) by activated CASP8. tBID can further translocate to mitochondria and cause MOMP through the activation of BAX and BAK1

The scientific observation of regulated cell death (RCD) historically began in 1842 when Karl Vogt noticed dying cells in toads. However, the surge in RCD research only started when the term “apoptosis” was coined in 1972 by John Kerr, Andrew Wyllie, and Alastair Currie^1^ (Fig. 1). Kerr et al. defined apoptosis as a form of programmed cell death (PCD) with morphological changes that differ from necrosis.^1^ Apoptosis and its dysregulation underlies various pathological and physiological processes, including cell homeostasis, tissue remodelling, and tumorigenesis.^2^ The identification of CED9 (also known as BCL2 in mammalian cells) and CED4 (also known as apoptotic peptidase-activating factor 1 [APAF1] in mammalian cells) from the studies of *Caenorhabditis elegans* development in the 1990s^3–5^ marks the beginning of an era of molecular apoptosis research that triggered the rapid expansion of RCD research. The molecular mechanisms regulating apoptosis have been extensively investigated in multiple organisms over the last 30 years. It is now established that apoptosis consists of two major subtypes, namely extrinsic and intrinsic apoptosis (Fig. 2). Extrinsic apoptosis is mediated by membrane receptors, especially by death receptors (e.g., fas cell surface death receptor [FAS, also known as CD95] and TNF receptor superfamily member 1A [TNFRSF1A, also known as TNFR1]), and is driven by initiator caspases CASP8 (also known as caspase 8) and CASP10 (also known as caspase 10).^6^ Alternatively, dependence receptors (e.g., unc-5 netrin receptor B [UNC5B, also known as UNC5H2] and DCC netrin 1 receptor [DCC]) may ignite extrinsic apoptosis via the activation of the initiator caspase CASP9 or dephosphorylation of death-associated protein kinase 1 (DAPK1, also known as DAPK) following the withdrawal of their ligands.^7^ In contrast, intrinsic apoptosis is ignited by mitochondrial outer membrane permeabilization (MOMP) that leads to the release of the mitochondrial proteins (e.g., cytochrome C, somatic [CYCS], diablo IAP-binding mitochondrial protein [DIABLO, also known as Smac], and HtrA serine peptidase 2 [HTRA2]) and subsequent activation of initiator caspase CASP9.^8^ MOMP is tightly controlled by the BCL2 family, including pro-apoptotic (e.g., BCL2 associated X, apoptosis regulator [BAX], BCL2 antagonist/killer 1 [BAK1, also known as BAK]), and anti-apoptotic (e.g., BCL2 and BCL2 like 1 [BCL2L1, also known as BCL-XL]) members.^2,9^ Although caspase activation does not guarantee cell death, CASP3, CASP6, and CASP7 are considered as important executioners due to their function in substrate cleavage and the destruction of subcellular structures^10,11^ (Box 1), culminating in the acquisition of the apoptotic morphotype.

Cell death may occur in multiple forms in response to different stresses, especially oxidative stress (Box 2). The loss of control over single or mixed types of cell death contributes to human diseases such as cancer, neurodegeneration, autoimmune diseases, and infectious diseases.^12,13^ During the past few decades, many novel forms of non-apoptotic RCD have been identified. In this review, we discuss our current understanding of the molecular machinery of each of the main types of non-apoptotic RCD, including necroptosis, pyroptosis, ferroptosis, entotic cell death, netotic cell death, parthanatos, lysosome-dependent cell death, autophagy-dependent cell death, alkaliptosis, and oxeiptosis, all of which can be inhibited by small-molecule compounds or drugs (Table 1). Finally, we describe the immunogenicity of cell death, which affects immune surveillance, inflammatory responses, tissue regeneration, and tumor therapy.

**Table 1:** Hallmarks of major types of RCD

Type	Morphological features	Biochemical features	Immune features	Major regulators	Major inhibitors (target)
Apoptosis	Cell rounding; nuclear condensation; membrane blebbing; apoptotic body formation	Activation of caspases; DNA fragmentation; ΔΨm dissipation; phosphatidylserine exposure	TCD or ICD	Positive: initiator caspase (CASP2, CASP8, CASP9, and CASP10); effector caspase (CASP3, CASP6, and CASP7); pro-apoptotic BCL2 family (e.g., BAK1, BAX, BOK, BCL2L11, BBC3, PMAIP1, and BID)^2,9^; TP53^264^	Z-VAD-FMK (pan caspase); emricasan (pan caspase); Q-VD-OPh (pan caspase); Z-VAD(OH)-FMK (pan caspase); Z-DEVD-FMK (CASP3, CASP6, CASP7, and CASP10); Z-VDVAD-FMK (CASP2); ivachtin (CASP3); Q-DEVD-OPh (CASP3); Ac-DEVD-CHO (CASP3 and CASP7); Z-IETD-FMK (CASP8); Q-LEHD-OPh (CASP9)
				Negative: anti-apoptotic BCL2 family (BCL2, BCL2L1, MCL1, BCL2L2, and BCL2L10)^2,9^	
Necroptosis	Cell swelling; rupture of plasma membrane; moderate chromatin condensation	Activation of RIPK1, RIPK3, and MLKL; cytosolic necrosome formation	ICD	Positive: RIPK1, RIPK3, and MLKL Negative: ESCRT-III, cIAPs, LUBAC, PPM1B, and AURKA	Necrostatin-1 (RIPK1); GSK872 (RIPK3); HS-1371 (RIPK3); necrosulfonamide (MLKL)
Pyroptosis	Lack of cell swelling; rupture of plasma membrane; bubbling; moderate chromatin condensation	Activation of CASP1, CASP3, and GSDMD; GSDMD cleavage; GSDMD-N–induced pore formation; IL1B release	ICD	Positive: CASP1, CASP11, and GSDMD Negative: GPX4, ESCRT-III, PKA	Ac-YVAD-cmk (CASP1); Z-YVAD (OMe)-FMK (CASP1); VX765 (CASP1); wedelolactone (CASP11); Ac-FLTD-CMK (GSDMD cleavage); MCC950 (NLRP3-inflammasome); isoliquiritigenin (NLRP3-inflammasome); glybenclamide (NLRP3-inflammasome); CY-09 (NLRP3-inflammasome); oridonin (NLRP3-inflammasome)
Ferroptosis	Smaller mitochondria; reduced mitochondria crista; elevated mitochondrial membrane densities; increased rupture of mitochondrial membrane	Iron accumulation; lipid peroxidation; ΔΨm dissipation; MAP1LC3B-I to MAP1LC3B-II conversion; glutaminolysis; caspase-independent	ICD	Positive: TFRC, ACSL4, LPCAT3, ALOX15, GLS2, DPP4, NCOA4, BAP1, BECN1, PEBP1, CARS,^122^ VDAC2/3, RAB7A, HSP90, and ALK4/5^265^ Negative: SLC7A11, GPX4, NFE2L2, HSPB1, HSPA5, FANCD2,^266^ NFS1,^267^ ITGA6,^268^ ITGB4,^268^ and OTUB1^269^ Dual: TP53	Deferoxamine (Fe); cyclipirox (Fe), deferiprone (Fe); ferrostatin-1 (ROS); liproxstatin-1 (ROS); β-mercaptoethanol (ROS); vitamin E (ROS); β-carotene (ROS); NAC (ROS); XJB-5-131 (ROS); zileuton (ROS); CoQ10 (ROS); baicalein (ROS); vildagliptin (DPP4); alogliptin (DPP4); linagliptin (DPP4); thiazolidinedione (ACSL4); rosiglitazone (ACSL4); selenium (GPX4)
Parthanatos	Chromatin condensation; large DNA fragmentation; lack of apoptotic body and small-scale DNA fragments; loss of membrane integrity; lack of cell swelling	Excessive activation of PARP1; ΔΨm dissipation; caspase-independent; NAD+ and ATP depletion; accumulation of poly ADP-ribose (PAR) polymers; AIFM1 release from mitochondria to nucleus	ICD	Positive: PARP1, AIFM1, MIF, and OGG1 Negative: ADPRHL2 and RNF146	BYK204165 (PARP1); AG-14361 (PARP1); iniparib (PARP1)
Entotic cell death	Cell-in-cell structure	Activation of adhesion proteins and actomyosin; LC3-associated phagocytosis	TCD or ICD	Positive: CDH1, CTNNA1, AMPK, RHOA, ROCK, myosin, ATG5, ATG7, PI3KC3, BECN1, CYBB, UVRAG, and RUBCN Negative: CDC42 and RNF146	C3-toxin (RHOA), Y-27632 (ROCK), blebbistatin (myosin)
Netotic cell death	Plasma membrane rupture; nuclear membrane collapse; chromatin fibre release	Formation of NETs; release and translocation of granular enzymes; histone citrullination	TCD or ICD	Positive: ELANE, MMP, MPO, CAMP/LL37, and PADI4	Tetrahydroisoquinolines (NETs); cl-amidine (PADI4); lactoferrin (NETs); DNase (NETs)
Lysosome-dependent cell death	Lysosome and plasma membrane rupture	Lysosomal membrane permeabilization; release of lysosomal hydrolytic enzymes; lysosomal iron-induced oxidative injury	ICD	Positive: cathepsins, STAT3, and TP53 Negative: NF-κB and MCOLN1	CA-074Me (CTSB); deferoxamine (Fe); NAC (ROS)
Autophagy-dependent cell death	Autophagic vacuolization	MAP1LC3B-I to MAP1LC3B-II conversion; increased autophagic flux and lysosomal activity	ICD	Positive: BECN1, Na^+^/K^+^-ATPase and AMPK Negative: mTOR	Chloroquine (lysosomal inhibitor); bafilomycin A1 (H^+^-ATPase inhibitor); concanamycin A (H^+^-ATPase inhibitor), 3-methyladenine (class III PI 3-kinase); spautin 1 (USP10 and USP13); wortmannin (PI 3-kinase)
Alkaliptosis	Necrosis-like morphology	Intracellular alkalinization; activation of NF-κB; caspase-independent	ICD	Positive: IKBKB and NF-κB Negative: CA9	NAC (pH), N-acetyl alanine acid (pH); IMD0354 (IKBKB); CAY10657 (IKBKB); SC514 (IKBKB)
Oxeiptosis	Apoptosis-like morphology	ROS-dependent; activation of KEAP1; NFE2L2-independent; caspase-independent; lack of nuclear translocation of AIFM1	TCD	Positive: KEAP1, PGAM5, and AIFM1	NAC (ROS)

Box 1 Caspases in cell death Caspases are a family of cysteine-dependent aspartate-specific proteases that play a critical role in the regulation of cell death, connecting to development, inflammation, and immunity.^10,11^ RCD is therefore categorized into two groups: caspase-dependent (e.g., apoptosis and pyroptosis) and caspase-independent RCD (e.g., necroptosis, ferroptosis, parthanatos, alkaliptosis, and oxeiptosis). In mammalian cells, caspases can be divided into four groups: initiator caspases (CASP2, CASP8, CASP9, and CASP10), effector caspases (CASP3, CASP6, and CASP7), inflammatory caspases (CASP1, CASP4, CASP5, CASP11, and CASP12), and the keratinization-relevant caspase (CASP14). Human CASP4 and CASP5 are functional orthologues of mouse CASP11 and CASP12, respectively. The mouse genome lacks CASP10.

Like many proteases, caspases initially exist as inactive zymogens, namely, procaspases. CASP8 and CASP10 have four domain structures, including the small subunit, large subunit, caspase activation and recruitment domain (CARD), and death effector domain (DED). CASP1, CASP2, CASP4, CASP5, CASP9, and CASP12 lack the DED motif, but contain other domains. In contrast, effector caspases (CASP3, CASP6, and CASP7) and CASP14 require cleavage by other caspases into small subunits and large subunits that assemble into active enzyme. These activated caspases can cleave substrates such as downstream caspases, cellular structural proteins, and immune molecules to cause cell death and inflammation. Caspases recognize at least four contiguous amino acids in their substrates, namely P4-P3-P2-P1. These substrates are cleaved by caspases after the C-terminal residue (P1), usually an Asp residue.

Initiator and effector caspases regulate apoptosis, whereas inflammatory caspases control pyroptosis. CASP3, CASP6, and CASP7 are essential executioner caspases in various types of apoptosis. They are usually activated by CASP8 and CASP9 in the extrinsic and intrinsic pathways, respectively. CASP8 coordinates the response to TNF in the induction of inflammation, apoptosis, and necroptosis. TNF is one of the most potent physiological inducers of the NF-κB pathway to transactivate genes coding for cytokines and pro-survival factors. This effect is achieved through the TNFRSF1A complex including FADD. Active CASP8 inactivates the TNFRSF1A complex activity by cleaving RIPK1, thus favoring the activation of CASP3 or CASP7 and subsequent apoptosis. In contrast, the inhibition of CASP8 by the pan-caspase inhibitor Z-VAD-FMK or genetic inactivation of either CASP8 or FADD leads to TNF-induced necroptosis via the activation of the RIPK1-RIPK3-MLKL pathway.^19,35^ CASP2 and CASP10 are alternative initiator caspases contributing to RCD under certain conditions, but the underlying mechanism remains unclear. CASP1, CASP4, CASP5, and CASP11 ignite pyroptosis by cleaving members of the gasdermin family, especially GSDMD, to induce pore formation and plasma membrane rupture.^82,99–103^ CASP12 is involved in endoplasmic reticulum stress-associated RCD^270^ (although this finding did not result in follow-up papers and has been disputed^271^) and functions as an anti-inflammatory regulator partly due to the inhibition of CASP1 inflammasome and the NF-κB pathway.^10,11^

Box 2 Oxidative stress in cell death Oxidative stress results from an imbalance between the production of ROS and the antioxidant capacity. ROS include superoxide anion (O_2_^•-^), hydroxyl radical (^•^OH), H_2_O_2_, and singlet oxygen (^1^O_2_). O_2_^•-^ is the one-electron reduction, whereas H_2_O_2_ is the two-electron reduction product of molecular oxygen. ^•^OH, a major initiator of lipoperoxidation, can be produced from iron-mediated Fenton reactions or high-energy ionizing radiation. ^1^O_2_ is an atypical ROS that is produced by the irradiation of molecular oxygen in the presence of photosensitizer pigments. Apart from mitochondria, other subcellular structures or organelles, including the plasma membrane, endoplasmic reticulum, and peroxisomes contribute to the production of ROS.

The antioxidant system may rely on enzymatic and non-enzymatic reactions. The enzymatic system comprises superoxide dismutase (SOD), catalase (CAT), glutathione peroxidase (GPX), and glutathione-S-transferase (GST). SOD isoenzymes, which include SOD1 in the cytoplasm and nucleus, SOD2 in mitochondria, and SOD3 in the extracellular space, catalyse the dismutation of O_2_^•-^ into either O_2_ or H_2_O_2._ CAT is mostly located in peroxisomes and is responsible for converting H_2_O_2_ into water and oxygen. GPX has eight members (GPX1-GPX8) in mitochondria, cytoplasm, and nuclei, and it functions to reduce lipid hydroperoxides to alcohols and to reduce H_2_O_2_ to H_2_O. The activity of GPX relies on the presence of the oligoelement selenium. GST detoxifies xenobiotic electrophilic substrates by conjugating them to reduced GSH. The major intracellular non-enzymatic antioxidants include GSH, metal-binding proteins, melatonin, bilirubin, and polyamines. GSH is considered as the most important endogenous antioxidant capable of directly interacting with ROS or electrophiles and by functioning as a cofactor for various enzymes, including GPX.

Oxidative damage is not only a cause, but also a consequence of various types of cell death. Excessive ROS can result in lipid peroxidation and damage to proteins and DNA. Peroxidation of membrane lipids not only leads to functional changes, but also causes structural damage, which finally results in cell rupture. Beyond its implication in apoptosis, lipid peroxidation is involved in various types of RCD such as ferroptosis,^117^ pyroptosis,^105^ necroptosis,^137^ autophagy-dependent death,^272^ parthanatos,^273^ and netotic cell death.^274^ DNA damage by oxidation is a major reason for genomic instability in the development of age-associated diseases. Apoptosis^13^ and parthanatos^180^ are usually associated with DNA damage. ROS may stimulate cell death pathways and trigger inflammation, resulting in inflammasome activation and pyroptosis.^275^ Therefore, the suppression of ROS could have crucial anti-inflammatory effects.

## Classification of cell death

Early classifications of cell death modalities depended on the morphological and structural details of individual tissues and cells. Accordingly, Schweichel and Merker published in 1973 a morphological hallmark system for classifying cell death into types I, II, and III in prenatal tissues treated with various embryotoxic substances.^14^ Type I cell death corresponds to apoptosis, and is characterized by cell shrinkage (pyknosis), membrane blebbing, apoptotic body formation, DNA fragmentation (karyorrhexis), and chromatin condensation. Apoptosis was also termed “shrinkage necrosis,” a form of nonpathologic cell death, by John Kerr in 1971.^15^ Type II cell death is often referred to as autophagy-dependent cell death, with the formation of large-scale autophagic vacuolization-containing cytosolic materials and organelles. Although there is no doubt that autophagy promotes cell survival in most cases,^16^ autophagy can also cause cell death, namely autophagy-dependent cell death, in specific circumstances.^17,18^ Type III cell death, namely necrosis, is characterized by the loss of membrane integrity and swelling of subcellular organelles (oncosis). Necrosis has long been considered as an uncontrolled type of cell death. In contrast, regulated types of necrosis such as necroptosis occur in a controlled manner.^12,13,19^

The current classification system of cell death has been updated by the Nomenclature Committee on Cell Death (NCCD), which formulates guidelines for the definition and interpretation of all aspects of cell death since 2005.^20^ The NCCD has released five position papers dealing with the classification of cell death (2005 and 2009),^20,21^ the molecular definitions of cell death subroutines (2012),^22^ essential versus accessory aspects of cell death (2015),^23^ and molecular mechanisms of cell death (2018).^24^ Currently, cell death can be fundamentally divided into accidental cell death (ACD) and RCD, based on functional aspects.^23^ ACD can be triggered by unexpected attack and injury that overwhelms any possible control mechanisms. In contrast, RCD involves precise signaling cascades, is executed by a set of defined effector molecules and has unique biochemical, functional, and immunological consequences (Table 1). RCD is also known as PCD when it occurs in physiological conditions.^23^ Based on its molecular characteristics, RCD can be classified into multiple subroutines, a few of which have clear physiological bearing (like necroptosis and pyropotosis, which are observed during development and/or in the context of viral infections) while others (like ferroptosis, entotic cell death, netotic cell death, parthanatos, lysosome-dependent cell death, autophagy-dependent cell death, alkaliptosis, and oxeiptosis) are less well-studied and may actually be limited to cellular responses to specific toxins that do not reflect normal physiology. Here, we adopt the viewpoint that cell death involves some kind of “regulation” (hence “RCD”) as long as specific genetic or pharmacological manipulations are able to interrupt the lethal cascade-causing cellular dismantling in response to external stimuli.

## Necroptosis

Necroptosis, a programmed form of necrosis showing morphological features similar to necrosis,^25^ was first observed in 1996 in pig kidney cells infected by the cowpox virus that expresses cytokine response modifier A (CrmA), a viral CASP1 and CASP8 inhibitor.^26^ In 1998, this observation was extended when L-M cells (a mouse fibroblast cell line) were found to be strongly sensitized to tumor necrosis factor (TNF, also known as TNFα)-induced necrotic cell death, suggesting that CASP8 negatively controls this type of cell death.^27^ Today it is known that necroptosis can be triggered by multiple stimuli, including the activation of death receptors (e.g., FAS and TNFRSF1A),^28^ toll-like receptors (e.g., toll-like receptor 3 [TLR3] and TLR4),^29^ nucleic acid sensors (e.g., Z-DNA–binding protein 1 [ZBP1, also known as DAI],^30^ retinoic acid receptor responder 3 [RARRES3, also known as RIG1],^31^ transmembrane protein 173 [TMEM173, also known as STING]^32,33^), and adhesion receptors.^34^ The same ligands (e.g., TNF, TNF superfamily member 10 [TNFSF10, also known as TRAIL], and Fas ligand [FASLG, also known as FasL or CD95L]) that ignite the extrinsic apoptosis pathway can trigger necroptosis when CASP8 activation at the death-inducing signaling complex (DISC) is prevented by means of caspase inhibitors (such as Z-VAD-FMK) or by the depletion of fas-associated via death domain (FADD).^35^

The era of molecular necroptosis research began in 2000 with the discovery of receptor-interacting serine/threonine kinase 1 (RIPK1) as a regulator of FASLG-induced necroptosis in T cells.^28^ RIPK1 is indeed a multifunctional signal kinase at the crossroads between inflammation, immunity, cell stress, cell survival, and cell death (Box 3). Subsequently, the identification of the pharmacological RIPK1 inhibitor necrostatin-1 led to the coining of the term “necroptosis.”^36,37^ Later, receptor-interacting serine/threonine kinase 3 (RIPK3) was unravelled as a downstream mediator of RIPK1 in death receptor-induced necroptosis.^38–40^ The subsequent discovery of mixed lineage kinase domain-like pseudokinase (MLKL) as the effector of necroptosis has largely enhanced our understanding of the molecular process of necroptosis.^41,42^

**Fig. 3:**
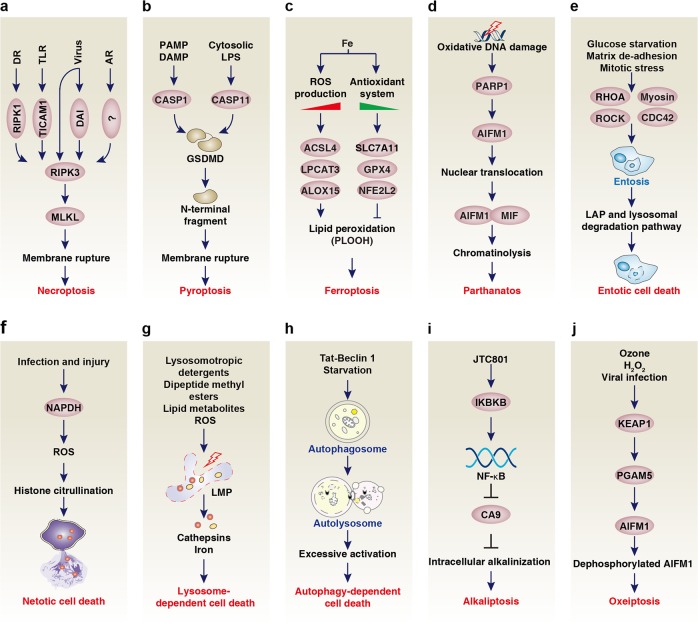
Core molecular mechanism of non-apoptotic regulated cell death. **a** RIPK3-stimulated MLKL is necessary for membrane rupture formation in necroptosis. Upstream elicitors include DR, TLR, and viruses, which induce RIPK3 activation through RIPK1, TICAM1, and ZBP1, respectively. In addition, RIPK3 is activated by AR via an unknown adaptor protein or kinases. **b** Pyroptosis is mostly driven by GSDMD after cleavage of this protein by CASP1 and CASP11 in response to PAMPs and DAMPs, or cytosolic LPS. **c** Ferroptosis is a form of cell death that relies on the balance between iron accumulation-induced ROS production and the antioxidant system during lipid peroxidation. The ACSL4-LPCAT3-ALOX15 pathway mediates lipid peroxidation. In contrast, several antioxidant systems, especially system xc^-^ that includes the core components SLC7A11, GPX4, and NFE2L2, inhibit this process. **d** Parthanatos is a PARP1-dependent form of cell death that relies on the AIFM1-MIF pathway. **e** Entotic cell death is a form of cellular cannibalism through the activation of entosis followed by the engulfing and killing of cells through LAP and the lysosomal degradation pathway. RHOA, ROCK, myosin, and CDC42 are required for entosis. **f** Netotic cell death is driven by NET release, which is regulated by NADPH oxidase-mediated ROS production and histone citrullination. **g** Lysosome-dependent cell death is mediated by releasing hydrolytic enzymes (cathepsins) or iron upon LMP. **h** Autophagy-dependent cell death is driven by the molecular machinery of autophagy. **i** Alkaliptosis is driven by intracellular alkalinization after IKBKB-NF-κB pathway-dependent downregulation of CA9. **j** Oxeiptosis is an oxygen radical-induced form of cell death driven by the activation of the KEAP1-PGAM5-AIFM1 pathway

An array of signaling pathways facilitate RIPK3 activation in several distinct ways (Fig. 3a) involving the homotypic interaction of RIP homotypic interaction motif (RHIM) domain-containing receptors, adaptors and kinases (ZBP1, toll-like receptor adaptor molecule 1 [TICAM1, also known as TRIF], RIPK1, and RIPK3). These RHIM domains of RIPK1 and RIPK3 mediate the formation of large hetero-amyloid signaling complexes that are initiated by different ligands.^43,44^ First, death receptor ligands induce the RHIM-mediated binding of RIPK1 to RIPK3, triggering the formation of specific signaling complexes, the “necrosomes,” ultimately resulting in MLKL activation.^38–40^ This process requires protein posttranslational modifications that are regulated by the ubiquitin ligase STIP1 homology and u-box containing protein 1 (STUB1, also known as CHIP),^45^ the aurora kinase A (AURKA),^46^ the protein phosphatase Mg^2+^/Mn^2+^-dependent 1B (PPM1B, also known as PP2CB),^47^ and the deubiquitinase TNF alpha-induced protein 3 (TNFAIP3, also known as A20).^48^ Second, TICAM1, but not RIPK1, is required for RIPK3-MLKL–dependent necroptosis in response to TLR ligands.^29^ Third, certain viruses can directly bind to RIPK3^49^ or promote the binding of the host protein ZBP1 to RIPK3 and subsequent MLKL activation.^30^ Fourth, RIPK3 activation by interferon alpha receptor or adhesion receptor occurs through an alternative pathway that does not require RIPK1, TICAM1 nor ZBP1.^34,50,51^

The phosphorylation of MLKL by RIPK3 at different residues in the C-terminal pseudokinase domain (S345/S347/T349 in mouse and S357/T358 in human) results in a conformational change and binding of inositolhexaphosphate (IP6) with positively charged patches in the N-terminal part of MLKL, followed by its recruitment to phosphatidylinosites, and insertion and multimerization in the plasma membrane, resulting in plasma membrane permeabilization.^41,42,52–56^ MLKL oligomerization and translocation to the plasma membrane can be enhanced by interactions with the molecular chaperone heat shock protein 90 alpha family class A member 1 (HSP90AA1, also known as HSP90)^57–60^ or by the local accumulation of inositol phosphates resulting from the activation of inositol phosphate kinase (e.g., inositol polyphosphate multikinase [IPMK] and inositol-tetrakisphosphate 1-kinase [ITPK1]).^61^ Strikingly, the endosomal sorting complexes required for transport (ESCRT)-III complex, a membrane scission machine, limits MLKL-mediated necroptosis and promotes membrane repair.^62^ MLKL has also been shown to regulate endosomal trafficking and extracellular vesicle generation.^63^ Thus, a fine balance between membrane injury and repair ultimately decides cell fate in necroptosis.

Early studies have revealed that mitochondrial events such as the production of mitochondrial reactive oxygen species (ROS),^64^ the activation of the mitochondrial phosphatase PGAM family member 5 (PGAM5, mitochondrial serine/threonine protein phosphatase), or the presence of a mitochondrial permeability transition may trigger necroptosis.^65^ How exactly mitochondrial ROS production contributes to necroptosis induction is still unsolved, but it may involve a redox sensing upstream of RIPK1 activation and RIPK3 recruitment.^66^ A connection between aerobic metabolism and necroptosis sensitivity might exist, as evidenced by RIPK3-mediated positive regulation of glutaminolysis and pyruvate dehydrogenase activity.^38,67^ However, other studies demonstrate that mitochondria are dispensable for necroptosis induced by death receptor signaling using PGAM5 knockdown cells.^55,68^ It has also been suggested that the formation of necrosomes with RIPK1, RIPK3, and MLKL in the nucleus may increase MLKL activity in plasma membranes.^69,70^ Moreover, nicotinamide adenine dinucleotide phosphate (NADPH) oxidase-derived ROS have been implicated in necroptosis in neutrophils.^71^ The functional significance of different ROS sources in necroptosis and how they impact the signal transduction remain to be further investigated.

In conclusion, RIPK3 and its substrate MLKL are necessary for necroptosis, whereas upstream RIPK1 contributes to this process in some cases (e.g., death receptor activation). RIPK3, independent of its kinase activity and independent of MLKL, also plays a regulatory role in apoptosis^72^ and in NLRP3-inflammasome activation and pyroptosis.^73^ Neither RIPK3 nor MLKL knockout mice show deficiency in embryogenesis, development, and homeostasis, suggesting no major role of necroptosis in nonchallenged conditions.^74,75^ A role for necroptosis in development and homeostasis is only revealed in conditions of FADD or CASP8 deficiency, demonstrating the important checkpoint function of CASP8 in controlling necroptosis in vivo.^74,75^ In contrast to the apparent absence of function during development and homeostasis, necroptosis is implicated in neurodegenerative diseases, chemotherapy responses, and tissue injury.^76^ Of note, data obtained from conditional knockout mice should be favoured over the use of systemic knockout mice that were generated using different sources of ES cells (129, C57BL/6J, or C57BL/6N) to avoid phenotypic interference of passenger mutations.^77^

Box 3 Regulation of RIPK1 in survival and cell death function When cells undergo cellular stress (endoplasmic reticulum stress, oxidative stress, DNA damage, pro-inflammatory stimuli) the default outcome is an adaptive response involving de novo expression of numerous genes and posttranslational modifications of target proteins (proteolysis, phosphorylation, and ubiquitylation) to maintain homeostasis or to induce cell death if the cellular stress remains unmitigated. RIPK1 is a central hub downstream of many cellular stress and immune receptor pathways such as TLR and TNF receptor family members, where it regulates the induction of pro-survival genes (e.g., BCL2, XIAP, and FLIP), inflammatory genes (cytokines and chemokines), and cell death through kinase-independent and kinase-dependent mechanisms.^276^ RIPK1 has two major faces. As a scaffold it recruits in an ubiquitylation-dependent way factors that initiate the activation of NF-κB and the MAPK cascade, and prevents CASP8-dependent apoptosis and RIPK3/MLKL-dependent necroptosis. As a kinase following its enzymatic activation, RIPK1 induces CASP8-mediated apoptosis and RIPK3/MLKL-mediated necroptosis. Transgenic knockin mice of kinase dead RIPK1 do not show a spontaneous phenotype but are resistant to TNF-induced systemic inflammatory response syndrome and show decreased pathogenesis in several inflammatory and degenerative diseases, suggesting that cell death may be an important etiologic factor in these pathologies.^72,277–279^ On the other hand, kinase dead RIPK1 knockin mice show increased sensitivity to infection, demonstrating the importance of RIPK1-driven cell death in immunosurveillance.^277,279,280^ What determines the switch between the RIPK1 scaffold and kinase functions that have such an impact on pathophysiological conditions? The most detailed insights into the regulation of these two opposing functions of RIPK1 were obtained from studying TNF-induced signaling pathways. TNF binding to TNFRSF1A causes in the first instance the formation of a receptor-associated complex I containing TRADD, RIPK1, and E3 ligases (TRAF2, cIAP1/2, and LUBAC), adding K63 and linear ubiquityl chains on RIPK1. This network of polyubiquityl chains forms a platform that recruits the IKK complex and MAP3K7/TAK1 complex controlling the NF-κB and MAPK signaling pathways and leading to pro-survival and pro-inflammatory gene induction.^281^ However, recently it was found that both IKK and MAPKAPK2/MK2 (activated by the TAK1/p38 MAPK axis) phosphorylate RIPK1 at distinct sites, preventing its catalytic autoactivation.^282–286^ When these phosphorylation-dependent brakes are absent, RIPK1 is recruited in complex II and will by default propagate apoptosis (complex IIb) or, in conditions of CASP8 deficiency, trigger RIPK1/RIPK3 necrosome formation and necroptosis. TBK1, a master integrator of stress and immune receptor signaling, also leads to the inactivation of RIPK1 kinase activity, suggesting that RIPK1 survival regulation goes beyond TNF signaling.^287^ As a consequence, conditions of the absence or inhibition of IAPs or LUBAC, absence of NEMO, inhibition of IKK, MAP3K7, or TBK1 strongly favor the catalytic autoactivation of RIPK1.^280^ It is hypothesized that pathological conditions, by regulating these survival checkpoints, may lead to enhanced sensitization of RIPK1-dependent apoptosis and necroptosis, contributing to inflammatory and degenerative diseases.^288^

## Pyroptosis

Pyroptosis is a form of RCD driven by the activation of inflammasome, a cytosolic multiprotein complex responsible for the release of interleukin (IL) 1 family members (e.g., interleukin-1β [IL1B] and IL18), the formation of ASC (apoptosis-associated speck-like protein containing a CARD, also called PYCARD or PYRIN and CARD domain-containing) specks, and the activation of pro-inflammatory caspases. The term pyroptosis was coined by Brad Cookson and coworkers^78^ to describe CASP1-dependent PCD in macrophages infected by *Salmonella* or *Shigella* and associated with the release of IL1B (IL1 was historically called leukocytic pyrogen, inspiring the name pyroptosis).^79,80^ CASP1 mediates the proteolytic processing of pro-IL1B and pro-IL18 into mature IL1B and IL18, respectively. This type of inflammatory cell death can be triggered by the activation of CASP1 or CASP11 in mice (the latter corresponding to CASP4 and CASP5 in humans) in macrophages, monocytes and other cells^81^ (Fig. 3b). Pyroptosis is morphologically distinct from apoptosis. Pyroptosis is characterized by the absence of DNA fragmentation in vitro, but by the presence of nuclear condensation coupled to cell swelling and the formation of large bubbles at the plasma membrane that eventually ruptures.^82,83^

The activation of inflammasomes in macrophages or monocytes requires two signals: a priming signal (that may be mediated by TLR ligands and IFN signaling) that induces the transcriptional upregulation of inflammasome components through nuclear factor of κB (NF-κB), and then a sensing signal (e.g., adenosine triphosphate [ATP] and lipopolysaccharide [LPS]) that triggers pro-inflammatory caspase-mediated pyroptosis. Inflammasomes that include canonical and noncanonical types can be activated in the context of infection, tissue injury, or metabolic imbalances.^81^ Canonical CASP1-dependent inflammasomes are divided into two subtypes, Nod-like receptors (NLR, e.g., NLR family pyrin domain-containing 1 [NLRP1], NLRP2, NLRP3, NLRP6, NLRP7, NLR family CARD domain-containing 4 [NLRC4]) and non-NLR (e.g., absent in melanoma 2 [AIM2]). They can be selectively activated by pathogen-associated molecular patterns (PAMPs), damage-associated molecular patterns (DAMPs), or other immune challenges. For example, NLRP3, the most intensively studied inflammasome, can be activated by a wide range of inflammatory stimuli such as bacterial peptidoglycans, extracellular ATP, and uric acid crystals, facilitated by the kinase NIMA-related kinase 7 (NEK7).^84^ The non-NLR inflammasome involving AIM2 is activated by cytosolic double-stranded DNA from bacteria or host cells.^85,86^ The CASP11-dependent noncanonical inflammasome is activated by cytosolic LPS from invading Gram-negative bacteria in macrophages, monocytes, or other cells.^87^ Lipid A moiety is required for cytosolic LPS binding to CASP11’s CARD domain, which causes CASP11 oligomerization.^88^ TLR4, a cell membrane receptor for LPS, is not required for cytosolic LPS-induced CASP11 activation.^89,90^ The cytoplasmic delivery of LPS requires the release of bacterial outer membrane vesicles (OMVs) by Gram-negative bacteria^91^ or the binding of LPS with high-mobility group Box 1 (HMGB1).^92^ The interplay between canonical (e.g., NLRP3- and AIM2-dependent) and noncanonical inflammasome pathways can amplify the inflammatory response and pyroptosis.^93,94^ Although eukaryotic translation initiation factor 2 alpha kinase 2 (EIF2AK2)/PKR and glycolysis may participate in CASP1-dependent inflammasome activation under certain conditions,^95–98^ their roles in CASP11 inflammasome remain unclear.

Several recent breakthroughs indicate that gasdermin D (GSDMD) is the key effector of pyroptosis^82,99–103^ (Fig. 3b). GSDMD is cleaved by CASP11 or CASP1 to produce a 22 kDa C- (GSDMD-C) and a 3l kDa N-terminal fragment (GSDMD-N).^101–103^ CASP11 auto-cleavage at the inter-subunit linker is essential for optimal catalytic activity and subsequent GSDMD cleavage.^104^ Once formed, GSDMD-N translocates to the inner leaflet of the plasma membrane and binds phospholipids, thus inducing the formation of pores that ultimately cause membrane lysis.^99^ In contrast, GSDMD-C inhibits GSDMD-N activity.^99,100^ While deficiency of the phospholipid hydroperoxidase glutathione peroxidase 4 (GPX4) in myeloid-derived cells increases CASP1- or CASP11-mediated GSDMD-N production, pyroptosis, and lethality after cecal ligation and puncture (CLP)-induced sepsis, the pharmacological inhibition of phospholipid hydrolysing enzyme phospholipase C gamma 1 (PLCG1) strongly protects against pyroptosis and CLP-induced septic death,^105^ indicating that lipid peroxidation promotes pyroptosis. Protein kinase A (PKA) is a major cyclic adenosine monophosphate (cAMP) effector to directly block CASP11-mediated GSDMD-N production in macrophages.^106^ Like necroptosis, ESCRT-III is also recruited to the plasma membrane to trigger membrane repair upon GSDMD activation.^107^ Other members of the gasdermin family (GSDMA, GSDMB, GSDMC, GSDME/DFNA5, and GSDMA3) have similar functions in membrane-disrupting cytotoxicity.^99^ It has been shown that following the blockage or deficiency of the bona fide pyroptosis pathway (ASC-CASP1/4), the induction of pyroptosis can be engaged through mechanisms such as CASP8-GSDMD^108,109^ and CASP3-GSDME,^110^ although the contribution of these alternative pathways to pyroptosis elicited by different triggers remains to be established in vivo.

Neutrophil elastase (ELANE), one of the antibacterial serine proteases, triggers GSDMD cleavage at a site that is closer to the N-terminus than the caspase cleavage site.^111^ Elastase-mediated GSDMD-N production induces neutrophil death as well as the formation of neutrophil extracellular traps (NETs) to intercept invading microorganisms.^112,113^ In addition, GSDMD-N can directly lyse bacteria (such as *Escherichia coli, Staphylococcus aureus*, and *Listeria monocytogenes*) after binding to cardiolipin and forming pores in their membranes.^100^ CASP1 and CASP11 also play a pyroptosis-independent role in antibacterial host defence. The formation of GSDMD pores can directly trigger IL1B secretion by macrophages before the cells undergo pyroptosis,^114^ indicating that distinct activation thresholds may control the active IL1B release by live cells and its passive shedding from dead cells once the cell explodes. The dynamics of pore formation and interaction with ion channels allow the existence of different stages and extents of plasma membrane permeabilization, resulting in the release of IL1B prior to spilling of DAMPs following full permeabilization.^115^

In summary, pyroptosis is a form of GSDMD-mediated RCD that plays a cell type-dependent role in inflammation and immunity. Of note, the first *Casp1*^*-/-*^ mice were established from 129 embryonic stem cells carrying an inactivating passenger mutation of the *Casp11* locus.^87^ Thus, the phenotype reported for *Casp1*^*-/-*^ mice actually results from deficiencies in both CASP1 and CASP11. Novel individual or combined transgenic mice are required to distinguish the contributions of CASP1 and CASP11 to pyroptotic signaling in a variety of different diseases that were studied in the past using the unintended double knockout.

## Ferroptosis

Ferroptosis is an iron- and lipotoxicity-dependent form of RCD. It was originally observed in 2003 using erastin (a cell-permeable compound from high-content screening) to selectively kill genetically-engineered cells with an oncogenic RAS mutation, but not normal cells^116^ In 2012, the term ferroptosis was formally used by Brent Stockwell to describe an iron-dependent form of non-apoptotic RCD induced by erastin.^117^ The morphology of erastin-induced ferroptotic cells is characterized by dysmorphic small mitochondria with decreased crista, as well as condensed, ruptured outer membranes,^117,118^ which might be under control of the pro-apoptotic BCL2 family members such as BH3-interacting domain death agonist (BID)^119^ and BCL2-binding component 3 (BBC3, also known as PUMA),^120^ but not BAX or BAK1.^117^ Mechanistically, these dying cells do not display any hallmarks of apoptosis or necroptosis.^117,118^ Instead, ferroptosis occurs via an iron-catalyzed process of lipid peroxidation initiated through non-enzymatic (Fenton reactions) and enzymatic mechanisms (lipoxygenases) (Fig. 3c). Polyunsaturated fatty acids (PUFAs) are the prime targets of lipid peroxidation of membranes.^121^ The deleterious effects of lipid peroxidation in ferroptosis execution can be neutralized by lipophilic radical traps such as vitamin E, ferrostatin-1, and liproxstatin-1.^117,118^ The mechanistic consequences of uncontrolled lipid peroxidation leading to ferroptotic cell death are still elusive. Using molecular dynamics models, it is hypothesized that membrane thinning and increased curvature drives a vicious cycle of access by oxidants, which ultimately destabilizes the membrane leading to pore and micelle formation.^122^ Additionally, lipid hydroperoxides decompose to reactive toxic aldehydes such as 4-hydroxy-2-nonenals or malondialdehydes, which may inactivate proteins through crosslinking^122^.

Essentially, ferroptosis can be induced in a canonical way by either inactivating GPX4, the major protective mechanism of biomembranes against peroxidation damage, or in a noncanonical way by increasing the labile iron pool. Two mechanisms have been described to inactivate GPX4: (1) an indirect way by the deprivation of the cofactor glutathione (GSH) through the depletion of the precursor Cys, as a result of the inhibition of the cystine/glutamate antiporter system xc^-117^ or the transsulfuration pathway,^123^ and (2) a direct way by binding and inactivating GPX4 by compounds such as RSL3, ML162, FINO_2_, withaferin A, or the FDA-approved anticancer agent altretamine.^121,124–129^ In addition, recent findings have proposed a noncanonical ferroptosis induction pathway upon iron overload using, for example, iron chloride, hemoglobin, hemin, or ferrous ammonium sulfate, which suffices to induce ferroptosis.^129,130^ CDGSH iron sulphur domain 1 (CISD1), a mitochondrial iron export protein, also inhibits ferroptosis by preventing mitochondrial iron accumulation and ROS production.^131^ The mitochondrial outer membrane proteins voltage-dependent anion channel 2 (VDAC2) and VDAC3 have been identified as direct targets of erastin that modulate mitochondrial function and contribute to ferroptosis.^132^ However, the contribution of mitochondria to ferroptosis remains controversial and may be context-dependent.^133^

System xc^-^ is composed of a regulatory subunit solute carrier family 3 member 2 (SLC3A2) and a catalytic subunit solute carrier family 7 member 11 (SLC7A11). This complex promotes the exchange of extracellular cystine and intracellular glutamate across the plasma membrane. Cystine in the cell is reduced to cysteine, which is required for the production of GSH. GPX4 uses GSH to eliminate the production of phospholipid hydroperoxides (PLOOH), the major mediator of chain reactions in lipoxygenases (Fig. 3c). System xc^-^ inhibitors (e.g., erastin, sulfasalazine, sorafenib, and glutamate) are considered as class I ferroptosis inducers (FINs), whereas direct GPX4 inhibitors are referred to class II FINs.^134^ Of note, GPX4 depletion was also shown to confer sensitivity to apoptosis,^135,136^ necroptosis,^137^ and pyroptosis.^105^ These findings suggest that lipid peroxidation can accelerate an array of distinct RCD modalities.

The likelihood of ferroptosis is determined by the balance between iron accumulation-induced ROS production and the antioxidant system that avoids lipid peroxidation (Fig. 3c). Increased iron uptake by transferrin receptor (TFRC, also known as TFR1) and reduced iron export by ferroportin favor oxidative damage and ferroptosis.^138^ Lipid peroxidation is influenced by several lipids and enzymes. Thus, the oxidation of PUFAs, including arachidonic acid (AA), by a catalytic pathway involving acyl-CoA synthetase long chain family member 4 (ACSL4), lysophosphatidylcholine acyltransferase 3 (LPCAT3), and arachidonate lipoxygenases (ALOXs, especially ALOX15) is required for lipotoxicity in ferroptosis.^121,139–142^ Phosphatidylethanolamine binding protein 1 (PEBP1, also known as RKIP), a scaffold protein inhibitor of protein kinase cascades, is required for the enzymatic activity of ALOX15 in ferroptosis.^142^ The upregulation of ACSL4, but not other ACSL members, seems to be a marker of ferroptosis.^141^ In addition to system xc^-^and GPX4, several integrated antioxidant and pro-survival proteins such as the transcription factor nuclear factor, erythroid 2 like 2 (NFE2L2, also known as NRF2)^143^ and certain heat shock proteins (HSPs),^144,145^ can inhibit lipid peroxidation in ferroptosis. In contrast, ROS generated during glutaminase 2 (GLS2)-mediated glutaminolysis may promote ferroptosis.^146^

NFE2L2 is a key transcription factor that regulates antioxidant defence or detoxification in the context of various stressors. NFE2L2-mediated transactivation of metallothionein 1G (MT1G, a cysteine-rich protein with a high affinity for divalent heavy metal ions), SLC7A11, and heme oxygenase 1 (HMOX1) limits ferroptosis.^143,147,148^ However, upon excessive activation of NRF2, HMOX1 gets hyperactivated and induces ferroptosis through increasing the labile iron pool upon metabolizing heme.^129,149,150^ Thus, the protective effect of HMOX1 is attributed to its antioxidant activity, while its toxic effect is mediated through the generation of ferrous iron that might boost Fenton-mediated decomposition of peroxides in case of insufficient buffering capacity by ferritin.

The tumor suppressor tumor protein p53 (TP53)^151^ and BRCA1-associated protein 1 (BAP1)^152^ can promote ferroptosis through the downregulation of SLC7A11 via transcriptional and epigenetic mechanisms, respectively. TP53 may also suppress ferroptosis by directly inhibiting the enzymatic activity of membrane-bound glycoprotein dipeptidyl peptidase 4 (DPP4, also known as CD26)^153^ or by increasing the expression of cell-cycle regulator cyclin-dependent kinase inhibitor 1A (CDKN1A, also known as p21).^154^ This has been observed in some cancers, in particular colorectal carcinoma, suggesting a context-dependent role of TP53 in the regulation of ferroptosis.^155^ An African-specific coding region variant of TP53, namely Pro47Ser, also affects ferroptosis senstivity and tumor supression.^156^

HSPs are a family of highly conserved molecular chaperones that are expressed in response to environmental stresses and render cells resistant to different types of cell death, including ferroptosis. In particular, heat shock protein family B [small] member (HSPB1, also known as HSP25 or HSP27)-mediated actin cytoskeleton protection inhibits ferroptosis via reducing iron uptake and subsequent oxidative injury.^144^ Heat shock protein family A [Hsp70] member 5 (HSPA5, also known as BIP or GRP78), an endoplasmic reticulum (ER)-sessile chaperone, binds and stabilizes GPX4, thus indirectly counteracting lipid peroxidation in ferroptosis.^145^ However, 2-amino-5-chloro-N,3-dimethylbenzamide (CDDO), an HSP90 inhibitor, can inhibit ferroptosis in cancer cells, indicating that HSP90 may play a different role in ferroptosis.^157^

The term “autophagy-dependent cell death” was originally used to describe cell death associated with autophagy based on morphological observation.^14^ It is now defined by the NCCD as a type of RCD that can be blocked by the suppression of autophagy.^24^ Recent findings indicate that ferroptosis induction is coupled to an increase in the turnover of lipidated microtubule-associated protein 1 light chain 3 beta (MAP1LC3B, also known as LC3, a marker of autophagosome) as well as the fusion of the autophagosome with lysosomes (namely, autolysosome formation, an important stage of autophagic flux), consistent with the notion that lipid oxidation stimulates autophagy.^158,159^ The genetic depletion of core autophagy effector molecules such as autophagy-related 5 (ATG5) and ATG7 block cell death by ferroptosis.^158,159^ Tat-Beclin 1, a strong direct inducer of autophagy, also enhances ferroptosis in cancer cells.^160^ The molecular mechanisms through which autophagy may contribute to ferroptotic demise may involve multiple pathways,^161^ such as the degradation of ferritin via nuclear receptor coactivator 4 (NCOA4)-dependent ferritinophagy (e.g., ferritin-specific autophagy),^158,159^ the inhibition of system xc^-^ activity via the formation of a BECN1-SLC7A11 protein complex,^160^ and the degradation of lipid droplets via ras-associated protein RAB7 (RAB7A)-dependent lipophagy.^162^ In addition, chaperone-mediated autophagy promotes GPX4 degradation and subsequent ferroptosis.^157^

In summary, ferroptosis is an non-apoptotic form of RCD driven by iron accumulation and lipid peroxidation, which can also involve autophagic processes, depending on the trigger.^163^ Excessive ferroptosis is likely to occur in certain human diseases, especially neurodegenerative and iron overload disorders, calling for its therapeutic suppression.^164^ In contrast, the induction of ferroptosis constitutes a potential strategy in cancer therapy.^164^ Note that almost 30 years ago a calcium-dependent non-apoptotic form of neuronal cell death, glutamate-induced toxicity, was coined as oxytosis that could be initiated by system xc^-^ inhibition and GSH depletion,^165,166^ and it was recently suggested that oxytosis and ferroptosis should be regarded as the same or at least a highly overlapping cell death pathway.^167^ That said, it remains to be determined whether ferroptosis is involved in “normal” physiology (e.g., development) or whether it only occurs in the context of pathologogical distortions (e.g., tissue injury) or pharmacological manipulations (e.g., anticancer therapy). Further evidence is required to understand this point. This general caveat applies to all modalities of RCD that are discussed below.

## Parthanatos

Parthanatos is a poly [ADP-Ribose] polymerase 1 (PARP1)-dependent RCD that is activated by oxidative stress-induced DNA damage and chromatinolysis (Fig. 3d). The term was coined by Valina and Ted Dawson in 2009.^168^ Unlike apoptosis, parthanatotic cell death occurs without the formation of an apoptotic body and small-size DNA fragments.^169^ Parthanatos also occurs in the absence of cell swelling, but is accompanied by plasma membrane rupture.^170^ PARP1 is a chromatin-associated nuclear protein that plays a critical role in the repair of DNA single-strand or double-strand breaks. PARP1 can recognize DNA breaks and use nicotinamide adenine dinucleotide (NAD^+^) and ATP to trigger poly (ADP-ribose)-sylation. The cleavage-mediated inactivation of PARP1 by caspases has been considered as a marker of apoptotic cell death.^171,172^ In contrast, 8-oxo-7,8-dihydroguanine, the common DNA base modification resulting from oxidative injury (e.g., ROS, ultraviolet light, and alkylating agents), triggers PARP1 hyperactivation.^173^ Hyperactivated PARP1 mediates parthanatos through at least two mechanisms, namely, the depletion of NAD^+^ and ATP (as it occurs during necrosis) and the dissipation of the mitochondrial inner transmembrane potential (an event commonly associated with apoptosis).^174^

Mechanistically, apoptosis-inducing factor mitochondria-associated 1 (AIFM1, also known as AIF),^175^ but not caspases and apoptotic DNase endonuclease G (ENDOG), is required for parthanatos execution.^176^ Hyperactive PARP1 binds AIFM1, which leads to AIFM1 release from mitochondria into the nucleus to produce parthanatotic chromatinolysis.^177^ This process can be negatively controlled by blocking PARP1 activity via the poly (ADP-ribose)-degrading protein ADP-ribosylhydrolase-like 2 (ADPRHL2, also known as ARH3)^178^ and the poly (ADP-ribose)-binding protein ring finger protein 146 (RNF146, also known as IDUNA),^179^ whereas it is positively regulated by enhancing PARP1 activity by the DNA glycosylase enzyme 8-oxoguanine DNA glycosylase (OGG1).^173^ More recently, macrophage migration inhibitory factor (MIF) has been identified as an AIFM1-binding protein with nuclease activity to produce large DNA fragments in the induction of parthanatos.^180^ AIFM1-independent parthanatos may also occur in some conditions such as dry macular degeneration.^181^ The interplay between AIFM1-dependent and -independent parthanatos with other RCDs such as autophagy-dependent cell death^182^ and necroptosis^183^ may be involved in various types of oxidative DNA damage-associated diseases, including neurodegenerative disease, myocardial infarction, and diabetes.

## Entotic cell death

Entotic cell death is a form of cell cannibalism in which one cell engulfs and kills another cell. The term entosis was coined in 2007 by Joan Brugge.^184^ Entosis and entotic cell death occur mostly in epithelial tumor cells in the contexts of aberrant proliferation,^184^ glucose starvation,^185^ matrix deadhesion,^184^ or mitotic stress.^186^ Entosis is characterized by the formation of cell-in-cell structures,^184^ which have also been observed in the urine and ascites from tumor patients.^187^ Entosis plays an ambiguous role in tumorigenesis, since it may trigger aneuploidy in engulfing cells^188^ and provide nutritional support for tumor growth,^185^ but may also mediate the removal of cancer cells by healthy neighbouring cells.^186^

Although their underlying mechanisms are not well-understood, cell adhesion and cytoskeletal rearrangement pathways play a central role in the control of entosis induction.^189,190^ The invasion of a live cell into a neighbouring cell during entosis requires the formation of adherent junctions, which is mediated by adhesion proteins cadherin 1 (CDH1, also known as E-cadherin) and catenin alpha 1 (CTNNA1), but not integrin receptors.^184,191,192^ Both intact actin and microtubules are required for cytoskeletal rearrangement during entosis. In particular, actomyosin, the actin-myosin complex in the cytoskeleton, is essential for the formation of cell-in-cell structures in entosis. The generation and activity of actomyosin is spatiotemporally controlled by ras homolog family member A (RHOA), rho-associated coiled-coil containing protein kinase (ROCK), and the myosin pathway^184,185,192–196^ (Fig. 3e). Consequently, pharmacologically targeting these core pathways by inhibitors such as C3-toxin, Y-27632, and blebbistatin diminishes entosis in vitro and in vivo.^189^

In addition to cell adhesion and cytoskeletal rearrangement pathways, other signaling molecules and regulators are also involved in the regulation of entosis through different mechanisms.^189^ For example, cell division cycle 42 (CDC42) depletion enhances changes in mitotic morphology and subsequent entosis.^186^ AURKA^197^ and the AMP-activated protein kinase (AMPK)^185^ promote entosis through the control of microtubule plasticity or energy metabolism, respectively. The chromatin-binding protein nuclear protein 1 (NUPR1, also known as P8), a transcriptional regulator, negatively regulates entosis through modulating AURKA activity or autophagy.^198^

The possible fates of the engulfed cells include cell division, release, or death. Entotic cell death involves the digestion of the engulfed cells by the host cells, which requires LC3-associated phagocytosis (LAP) and the cathepsin B (CTSB)-mediated lysosomal degradation pathway^189,190^ (Fig. 3e). However, entosis does not involve apoptosis effector caspases and is not regulated by proteins of the BCL2 family.^189,199^ LAP bridges phagocytosis and autophagy; this process is regulated by the core LC3 lipidation machinery (e.g., ATG5, ATG7, class III phosphatidylinositol 3-kinase complex [e.g., phosphatidylinositol 3-kinase catalytic subunit type 3 (PI3KC3), also known as VPS34], phosphoinositide-3-kinase regulatory subunit 4 [PIK3R4, also known as VPS15], and BECN1), cytochrome B-245 beta chain (CYBB, also known as NOX2)-mediated ROS production, other autophagy regulators (e.g., UV radiation resistance-associated [UVRAG] and RUN domain and cysteine-rich domain-containing beclin 1 interacting protein [RUBCN, also known as Rubicon]).^200^ Entosis is often observed in neoplasia and its frequency correlates with tumor stage, calling for a further in-depth evaluation of the possibility of targeting this phenomenon. However, at this stage, there are no reagents available that would allow us to inhibit or induce entosis in a selective fashion, i.e., without influencing other cell death modalities.

## Netotic cell death

Netotic cell death is a form of RCD driven by NET release. NETs are extracellular net-like DNA-protein structures released by cells in response to infection or injury. NET formation and release, or NETosis, was first observed in neutrophils upon exposure to phorbol myristate acetate or IL8 by Arturo Zychlinsky’s lab in 2004.^201^ NETs can also be generated by other leukocyte populations (e.g., mast cells, eosinophils, and basophils), epithelial cells, and cancer cells in response to various stresses.^202–204^ Elevated NETosis not only acts against the spread of infection by trapping pathogenic microorganisms (e.g., bacteria and viruses), but also promotes DAMP release, thus possibly contributing to the pathogenesis of autoimmune disorders (e.g., systemic lupus erythematosus, rheumatoid arthritis, asthma, vessel vasculitis, and psoriasis), ischemia-reperfusion injury, and tumor development.^205^ A recent study indicates that inflammation-associated NET production can awaken nearby dormant cancer cells to redivide.^206^ This effect may rely on the degradation of laminins, a major adhesive component of basement membranes^206^; however, this needs further mechanistic exploration.

NETosis is a dynamic process and relies on multiple signals and steps including NADPH oxidase-mediated ROS production, autophagy, the release and translocation of granular enzymes (e.g., elastase, neutrophil expressed [ELANE], matrix metalloproteinase [MMP], and myeloperoxidase [MPO]) and peptides from the cathelecidin family (e.g., cathelicidin antimicrobial peptide [CAMP, also known as LL37]) from the cytosol to the nucleus.^207,208^ This is followed by histone citrullination, favoring chromatin decondensation, the destruction of the nuclear envelope, and the release of chromatin fibres^209^ (Fig. 3f). Peptidyl arginine deiminase 4 (PADI4, also known as PAD4) is the enzyme responsible for catalyzing the conversion of arginine to citrullin residues in histones.^210^ A recently discovered pathway of PADI4-independent NETosis^211^ may occur downstream of death signals that are normally involved in other types of RCD such as pyroptosis, necroptosis, and autophagy-dependent cell death.^212^ The alterations of cell surface associated to netotic cell death are initiated by entropic swelling of chromatin through a yet elusive mechanism.^213^ Of note, lactoferrin, a component of neutrophil granules, can block netotic cell death and inflammation both in vitro and in vivo.^214^ In addition to the key role of GSDMD in pyroptosis, GSDMD is also involved in the induction of NETosis to digest the pathogen,^112,113^ indicating a crosstalk between pyroptosis and NETosis pathways in the innate immunity.

## Lysosome-dependent cell death

Lysosome-dependent cell death (LCD), also known as lysosomal cell death, is a type of RCD mediated by hydrolytic enzymes that are released into the cytosol after lysosomal membrane permeabilization (Fig. 3g).^215^ The idea of LCD was first expressed by Christian de Duve, who discovered lysosomes as the cellular degradation machinery in 1955, and the term “lysosomal cell death” was coined in 2000.^216^ Lysosomes are acidic cellular organelles that can degrade a variety of heterophagic and autophagic cargos, including unused intracellular macromolecules (nucleic acids, proteins, lipids, and carbohydrates), entire organelles (e.g., mitochondria), and invading pathogens.

Lysosomes become leaky when cells are exposed to lysosomotropic detergents (e.g., O-methyl-serine dodecylamide hydrochloride), dipeptide methyl esters (e.g., Leu-Leu-OMe), lipid metabolites (e.g., sphingosine and phosphatidic acid), and ROS.^215^ Lysosomal membrane permeabilization may also amplify or initiate cell death signaling in the context of apoptosis, autophagy-dependent cell death, and ferroptosis.^217,218^

Among lysosomal hydrolases, cathepsins (a large family of cysteine peptidases) play a major role in LCD. Different cathepsins are responsible for the initiation and execution of LCD, depending on the context of lysosomal membrane permeabilization.^219^ The transcription factors signal transducer and activator of transcription 3 (STAT3)^220^ and TP53^221^ may favor LCD induction through the selective upregulation of cathepsins (e.g., CTSB, cathepsin L [CTSL] and cathepsin D [CTSD]) expression. In contrast, the NF-κB–elicited expression of serpin family A member 3 (SERPINA3) results in the inhibition of CTSB and CTSL.^222^ Moreover, the suppression of mucolipin 1, an ion channel in the lysosome (MCOLN1, also known as TRPML1) results in a lysosomal trafficking defect, which promotes CTSB release and consequent LCD.^223^

Blocking cathepsin expression or activity can block LCD. However, cathepsins are not the sole effectors of LCD because lysosomes store abundant iron, meaning that lysosomal membrane permeabilization can result in the release of this toxic metal into the cytosol,^224^ thus contributing to ferroptosis.^218,225^ Impaired lysosomal degradation and the LCD pathway are associated with increased oxidative injury and contribute to lysosomal storage disorders and age-associated diseases.^226,227^ Based on the cellular context, LCD can adopt necrotic, apoptotic, autophagic, or ferroptotic-like features, adding complexity to this cell death pathway.^215,217,218^

## Autophagy-dependent cell death

Autophagy-dependent cell death is a type of RCD driven by the molecular machinery of autophagy (Fig. 3h). Macroautophagy (hereafter called “autophagy”) is an evolutionarily conserved degradation pathway and has been implicated in human disease and aging.^228,229^ The process of autophagy involves the sequential formation of three unique membrane structures, namely the phagophore, autophagosome, and autolysosome. Over 40 autophagy-related genes/proteins (ATGs) play key roles in autophagic membrane dynamics and processes.^230^

As a dynamic recycling system, the bulk and nonselective autophagy process is generally considered as a pro-survival mechanism in response to multiple types of cellular stresses.^16^ Nevertheless, autophagy can selectively degrade pro-survival proteins related to other types of RCD, thereby tipping the balance from life to death.^231–234^ Ferritinophagy causes ferroptosis due to the selective degradation of ferritin (an iron storage protein), consequently causing iron release and oxidative injury.^158,159^ The degradation of protein tyrosine phosphatase, nonreceptor type 13 (PTPN13, a negative regulator of extrinsic apoptosis)^235^ favors apoptosis, while autophagic digestion of baculoregulator repeat containing 2 (BIRC2, also known as cIAP1, a negative regulator of necroptosis)^236^ facilitates the ignition of necroptosis.

In 2013, Beth Levine described “autosis” as a subtype of autophagy-dependent cell death induced by nutrient deprivation or by Tat-Beclin 1, an autophagy-inducing peptide fusing amino acids from BECN1 and HIV Tat protein.^17^ Autosis is morphologically characterized by enhanced cell-substrate adherence, fragmented or vanished ER structure, focal swelling of the perinuclear space, and mild chromatin condensation.^17^ At the molecular level, Tat-Beclin 1–induced autosis can be inhibited by blocking upstream Na^+^/K^+^-ATPase, a plasma pump linking ion homeostasis and ER stress.^17^ Interestingly, iron overload stimulates Na^+^/K^+^-ATPase activity in the human erythrocyte membrane,^237^ which may lead to ferroptosis. However, the exact relationship between autosis and ferroptosis remains to be determined.

Autophagy-dependent cell death probably plays a pathogenic role in neurotoxicity and hypoxia-ischemia-induced neuronal death,^232–234^ indicating that this type of RCD can possibly be targeted for neuroprotection.

## Alkaliptosis

Alkaliptosis is a novel type of RCD driven by intracellular alkalinisation.^238^ The word “alkaliptosis” was termed in 2018 by our group.^238^ A screen of small-molecule compound library targeting G-protein coupled receptors (GPCR) for cytotoxic activity on a human pancreatic cancer cell line led to the idenfication of JTC801. The latter is an opioid analgesic drug that efficiently kills a panel of human pancreas, kidney, prostate, skin, and brain cancer cell lines,^238^ and these cytotoxic effects were not related to apoptosis, necroptosis, autophagy, or ferroptosis, because genetically or pharmacologically blocking these forms of RCD failed to reverse JTC801-induced cell death.^238^ In contrast, the inhibition of intracellular alkalinization by N-acetyl cysteine, N-acetyl alanine acid, and acidic culture media blocked JTC801-induced cell death.^238^ At the molecular levels, alkaliptosis requires inhibitor of nuclear factor kappa B kinase subunit beta (IKBKB, also known as IKKβ)-NF-κB pathway-dependent downregulation of carbonic anhydrase 9 (CA9), an enzyme participating in pH regulation (Fig. 3i). Opioid-related nociceptin receptor 1 (OPRL1), the target that accounts for the analgesic activity of JTC801, is dispensable for alkaliptosis.^238^ Of note, JTC801 has also been reported to induce apoptosis in human osteosarcoma cells,^239^ suggesting that the type of RCD triggered by JTC801 depends on the cellular context.

It should be noted that the pathological significance of alkaliptosis in human disease remains fully elusive although metabolic alkalosis is a unique acid-base disorder with kidney or lung injury.^240^ The significance of the core effector molecules of alkaliptosis also remains unclear.

## Oxeiptosis

Oxeiptosis is a novel oxygen radical-induced caspase-independent RCD driven by the activation of the KEAP1-PGAM5-AIFM1 pathway (Fig. 3j).^241^ This term was introduced in 2018 by Andreas Pichlmair’s lab in a study reporting on the response of mice to ozone and that of cultured fibroblasts and epithelial cells to hydrogen peroxide (H_2_O_2_).^241^ Ozone- or H_2_O_2_-induced oxeiptosis is independent of apoptotic or pyroptotic caspases, necroptosis, autophagy, and ferroptosis.^241^ The KEAP1-NFE2L2 pathway has been known to mediate cytoprotective responses to oxidative injury.^241^ However, hyperactivated KEAP1 can mediate H_2_O_2_-induced oxeiptosis in an NFE2L2-independent manner,^241^ through a pathway that involves KEAP1 interaction partner PGAM5, a mitochondrial serine-threonine phosphatase that dephosphorylates AIFM1 at Ser116.^241^ Unlike AIFM1-mediated caspase-independent apoptosis and parthanatos, dephosphorylated AIFM1-mediated oxeiptosis does not require the translocation of AIFM1 from mitochondria to the nucleus.^241^ In vivo, *Pgam5*^*–/–*^ mice are more sensitive to inflammation and injury following ozone treatment or viral infection, indicating that oxeiptosis may suppress inflammation.^241^ However, it remains an open conundrum how H_2_O_2_ may induce so many different cell death modalities including oxeiptosis,^241^ apoptosis,^242^ necrosis,^242^ and ferroptosis.^243^ Understanding the location- and modification-dependent role of AIFM1 may help us to distinguish these different types of RCD. The role of oxeiptosis in pathological cell death in human diseases also remains largely unknown.

## Immunological consequences of cell death

Cell death induced by stimuli may occur in a way that the immune system is alerted, triggering immunity against dead-cell antigens. This “immunogenic cell death” (ICD), a term coined in 2005,^244^ contrasts with silent efferocytosis, in which dying and dead cells are cleared by phagocytosis without any inflammatory or immune reaction, as well as with tolerogenic cell death (TCD) that actively inhibits immune responses.^245^ Although apoptosis has generally been considered as a TCD, accumulating evidence suggests that apoptosis can be an ICD when induced under certain conditions.^246^ An acute or chronic inflammatory response elicited by dying cells not only promotes tissue regeneration and limits infection, but may also cause tissue injury and disease.^247^ Given the fundamental role of inflammation in a variety of human diseases, it is important to understand the key mediators and pathways that drive this response.

**Fig. 4:**
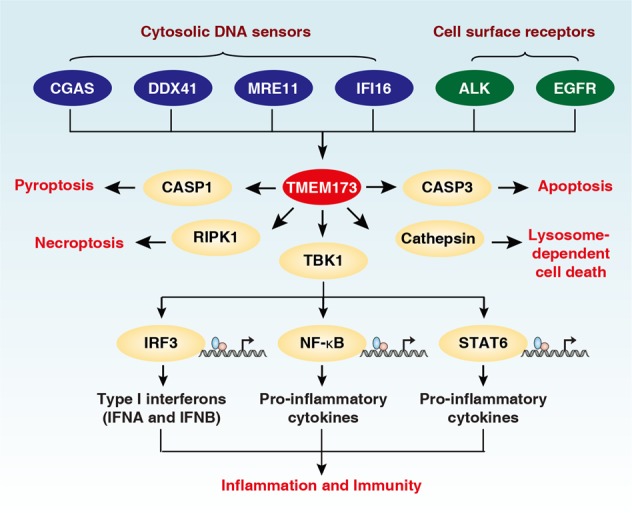
Central role of TMEM173 in inflammation, immunity, and cell death. TMEM173 can be activated by cytosolic DNA sensors (e.g., CGAS, DDX41, MRE11, and IFI16), or cell surface receptors (e.g., ALK and EGFR) in response to various DNAs from the pathogen and host. The activation of STING not only promotes inflammation and the immune response through TBK1-mediated transcription factor activation, but also ignites various cell death pathways, including apoptosis, necroptosis, pyroptosis, and lysosome-dependent cell death

There is no doubt that the release of DAMPs by dead or dying cells is an important factor regulating the balance between ICD and TCD. The immune system can recognize two types of danger signals.^248^ PAMPs from microbes are recognized by pattern recognition receptors (PRRs). Endogenous DAMPs, which act on the same PRRs as the PAMPs, can be proteins (e.g., HMGB1, histones, and transcription factor A, mitochondrial [TFAM]) and nonproteaceous entities (e.g., DNA, RNA, and extracellular ATP). The release of DAMPs is a hallmark of various types of cell death, although they may exhibit distinct expression profiles in response to different stimuli.^249^ DAMPs activate different PRRs, such as TLRs, advanced glycosylation end-product specific receptor (AGER, also known as RAGE), and DNA sensors (Box 4) that are widely expressed in leukocytes and other cell types.^248^ A number of inflammation-related pathways, involving for example the RIPK1-NF-κB,^250^ DNA-TMEM173 (Fig. 4),^251^ and IL-17A–IL-17R^252^ pathways have been documented to mediate the ICD-associated immune response. However, another study suggests that the immunogenicity of necroptotic cells does not correlate with the activation of the RIPK3-RIPK1-NF-κB pathway.^253^

The pathophysiological role of ICD has amply been documented in the context of chemotherapy-induced anticancer immune responses.^244^ Several cytotoxic antineoplastics stimulate the immune system by stressing and killing cancer cells in a way that results in the exposure of DAMPs such as calreticulin^246^ at the surface or the release of DAMPs such as ATP,^254^ annexin A1,^255^ HMGB1,^256^ and TFAM^257^ into the extracellular space. ICD may occur in the context of apoptosis and necroptosis, meaning that the immunological consequences are not tied to the cell death modality itself but rather to the exposure/release of DAMPs that may occur as a consequence of premortem stress responses.^247^ Thus, calreticulin exposure is tied to a partial endoplasmic reticulum stress response,^246^ while ATP release occurs through an autophagy-dependent mechanism.^254^ ICD of malignant cells favors the cross-presentation of tumor-associated antigens by dendritic cells, resulting in the induction of cytotoxic T lymphocytes that then play an essential role in keeping tumors in check.^247^ In addition to cancer, ICD is also implicated in infectious disease.^247^

It is interesting to note that the redox status of DAMPs may affect their immune activity as this has been exemplified for HMGB1, a protein that is usually present in the nucleus yet can translocate to the cytoplasm and undergo cell death-associated release. Extracellular HMGB1 remarkably initiates, amplifies, and perpetuates the inflammatory response if it is nonoxidized.^258^ However, the oxidized form of HMGB1 favors the induction of immune tolerance in antigen-presenting cells^259^ and may also promote the expression of immune checkpoint molecules (such as CD274, also known as PD-L1) to limit anticancer immunity.^260^ The proteolytic cleavage or degradation of HMGB1 also limits its immunostimulatory activity under some conditions.^261,262^ Thus, HMGB1 may act as a tightly controlled universal DAMP that mediates both ICD and TCD depending on its abundance and oxidation status.^263^

In spite of the wealth of information on the rules governing the immunological consequences of cell death, a systematic exploration of non-apoptotic RCD subroutines with respect to their immunogenicity is still elusive.

Box 4 DNA sensors in cell death The release of genomic or mitochondrial DNA into the cytoplasm or into the extracellular space is a hallmark of RCD.^289^ Emerging evidence has revealed that TMEM173 (Fig. 4), AIM2, and ZBP1 are major DNA-sensing pathways in the regulation of inflammatory and immune responses. TMEM173 is an endoplasmic reticulum protein and recognizes various DNA products from bacteria, viruses, and dead or dying host cells through both CGAS (also known as cGAS)-dependent and -independent pathways.^290–292^ In addition, the activation of other cytosolic nucleic acid sensors (DDX41, MRE11, IFI16, and ZBP1)^293–296^ as well as membrane receptors (ALK receptor tyrosine kinase [ALK] and epidermal growth factor receptor [EGFR])^297^ can function as upstream signals to initiate TMEM173 activation in response to xenogenic DNA from pathogens or ectopic DNA from the host. Mechanistically, TMEM173 binds to TBK1 and then triggers the activation of transcription factors such as interferon regulatory factor 3 (IRF3), NF-κB, and signal transducer and activator of transcription 6 (STAT6), thus promoting type I IFN and cytokine production and the consequent inflammation and immune responses.^298^ TMEM173 knockout mice are resistant to lethal infection,^297^ sterile inflammation,^299,300^ and inflammation-driven carcinogenesis and tumor metastasis,^301^ as well as to inflammation-driven age-associated diseases.^302^ At least in some settings, the excessive activation of TMEM173 in T lymphocytes and myeloid cells can cause apoptosis, necroptosis, pyroptosis or LCD, although the role of TBK1 in these settings remains unidentified.^303–305^ Moreover, TMEM173 contributes to the ICD-mediated antitumor immune response.^251,306^ These observations point to TMEM173 as an important DNA sensor that acts both in immune and non-immune cells.

AIM2 was originally identified as a receptor of pathogen double-stranded DNA from *Francisella*, *Listeria*, *Mycobacterium*, mouse cytomegalovirus, vaccinia virus, *Aspergillus*, and *Plasmodium* species. AIM2 may also detect cytoplasmic or nuclear self-DNA from necrotic cells for inflammation activation and pyroptosis, thus contributing to autoimmune and inflammatory diseases such as dermatitis, arthritis, pancreatitis and radiation-induced inflammation.^307^ AIM2 may promote or limit tumor development in a cancer type-dependent fashion.^260,308^

ZBP1 acts as a cytosolic sensor for viral DNA or RNA and stimulates inflammatory and immune response through the activation of RIPK3-MLKL–dependent necroptosis, as well as the TMEM173 pathway.^309^ Nonetheless, the role of ZBP1 in tumor immunity remains unclear.

## Conclusions and perspectives

RCD occurs through a variety of subroutines that cause cells to be dismantled in different ways, hence producing distinct morphological changes and immunological consequences. In spite of this “biodiversity,” the evolutionary relationship between distinct RCD pathways remains unknown. Oxidative stress can lead to various types of RCD, while the sources of ROS as well as the efficacy of antioxidant defences are context-dependent. It will be important to assemble a standard panel of biomarkers and functional tests including genetic and pharmacological inhibition studies to accurately distinguish between different forms of RCD that may occur in a “pure” form or in “mixed” variants, in which distinct lethal subroutines come into action in a parallel and sometimes hierarchized fashion. It is well known that the suppression of apoptosis by caspase inhibition may reveal necroptotic pathways, and similar backup systems might come into action when other cell death modalities are inhibited. It is plausible that RCD does not only play a housekeeping role in maintaining organismal homeostasis, and that it may play a major role in unwarranted cellular demise. The release of DAMPs during RCD provides potent signals to stimulate local inflammatory or systemic immune responses. The development of novel drugs for selectively intercepting (or, in sharp contrast, activating) the RCD pathway holds great promise for preventing and treating human diseases in which cell loss must be avoided (or when the elimination of malignant cells is a therapeutic goal). More research on RCD is needed to define the interplay between distinct cell death signaling pathways, identify unique molecular effectors for each type of RCD, and evaluate pro-survival or reprogramming mechanisms against RCD. In addition, more research is needed to define the critical point of no return of each RCD subroutine and to investigate the role of excessive or deficient RCD in human disease.
